# Coil Sketching for Fast and Efficient 4D Lung MRI Reconstruction

**DOI:** 10.1002/mrm.70169

**Published:** 2025-11-07

**Authors:** Joseph W. Plummer, Pierre Daudé, Anastasia Tsakirellis, Jordan Taylor, Joel Moss, Rajiv Ramasawmy, Ahsan Javed, Adrienne E. Campbell‐Washburn

**Affiliations:** ^1^ Cardiovascular Branch, Division of Intramural Research, National Heart, Lung, and Blood Institute National Institutes of Health Bethesda Maryland USA; ^2^ Critical Care Medicine and Pulmonary Branch, Division of Intramural Research, National Heart, Lung, and Blood Institute National Institutes of Health Bethesda Maryland USA

**Keywords:** 4D, coil sketching, low rank reconstruction, lung MRI, motion‐compensation, non‐Cartesian, respiratory‐resolved, Toeplitz

## Abstract

**Purpose:**

To develop and evaluate a memory‐efficient and accelerated reconstruction framework for respiratory‐resolved 4D lung MRI using coil sketching and Toeplitz approximation, enabling high‐quality motion‐compensated low‐rank (MoCo‐LR) reconstructions on clinically accessible GPU hardware.

**Theory and Methods:**

Respiratory‐resolved 4D MRI enables non‐invasive assessment of pulmonary structure and function but is limited by computational and memory demands from large matrices and long acquisitions. We extend the coil sketching framework—previously proposed for 3D imaging—to 4D (3D + time), allowing the data consistency term of compressed‐sensing objective functions to be solved with reduced GPU memory consumption and reconstruction duration while preserving image accuracy. Additionally, we implement Toeplitz approximation to accelerate repeated applications of the normal encoding operator, further reducing computational demand. Finally, we outline a MoCo‐LR regularization technique that uses only forward deformations, improving reconstruction stability over many iterations. Reconstructions were performed across several regularization schemes using a 3D stack‐of‐spirals acquisition and evaluated for memory footprint, speed, and image accuracy.

**Results:**

The proposed 4D coil sketching method reduced memory usage by ˜3‐fold compared to fully sampled reconstructions and enabled high‐resolution MoCo‐LR reconstructions in < 10 min on < 48 GB GPUs. Toeplitz approximation further reduced runtime with minimal impact on image quality. Compared to conventional coil compression, coil sketching preserved parenchymal signal and structural fidelity in low‐SNR regions.

**Conclusion:**

Coil sketching and Toeplitz approximation provide a generalizable, hardware‐efficient solution for 4D lung MRI reconstruction. These methods reduce computational barriers, improve reconstruction speed, and maintain image quality, offering a path toward broader clinical adoption of respiratory‐resolved lung MRI.

## 1 Introduction

Respiratory‐resolved 4D MRI (3D + time) of the lungs is a powerful technique that enables simultaneous assessment of pulmonary structure and function. Data are typically acquired using free‐breathing, non‐Cartesian ultra‐short echo time (UTE) sequences, which offer robustness to motion and are well‐suited for imaging tissues with short $T_{2}^{*}$ [1, 2]. For respiratory‐resolved imaging, raw data are retrospectively sorted into multiple respiratory states, then reconstructed using iterative regularized reconstructions to generate aliasing‐free images across the breathing cycle [3].

These images not only provide clinicians a useful view of lung structure but also can be used to quantify respiratory‐induced regional lung density changes—an approximate surrogate for ventilation [4, 5, 6]. Such measurements are the driving force behind functional lung imaging techniques, such as Fourier decomposition [4], voxel‐wise lung ventilation (VOLVE) [7], and phase‐resolved functional lung imaging (PREFUL) [8]. These techniques are appealing due to their simplicity and minimal reliance on specialized hardware, which has enabled widespread demonstration across various diseases, including COVID‐19, asthma, and lymphangioleiomyomatosis (LAM) [9, 10, 11].

However, a major limitation of respiratory‐resolved lung MRI, particularly at high resolution or in 3D, is image reconstruction complexity. Very‐large matrix sizes and number of respiratory phases contribute to long reconstruction times (hours), large memory footprint (> 60 GB), and ultimately, reduced clinical adoption.

Graphical processing unit (GPU) computing can address reconstruction speed but requires extensive GPU memory capacity which can cost beyond the means of most clinical and research sites. Strategies to reduce GPU memory include batched reconstructions, where reconstruction tasks are divided into smaller sub‐problems composed of multiple small subsets of coils, or coil compression [12], where a representative smaller number of virtual coils are used to compress the k‐space data. While coil compression is commonly used, it comes at the cost of image accuracy in low signal regions [13], which are particularly prevalent in the lungs.

Recently, a “coil sketching” technique was proposed to reduce large compressed‐sensing problems into smaller subproblems comprised of random subsets of MRI coils [14]. This approach was demonstrated for 3D non‐Cartesian wavelet and total‐variation reconstructions of the liver, where it significantly reduced GPU memory footprint and reconstruction time. Critically, coil sketching preserved image accuracy and SNR in low signal regions where coil compression was inadequate.

In this study, we expand the coil‐sketching framework into 4D (3D + time) reconstructions. Furthermore, we demonstrate its efficacy at solving motion‐compensated low‐rank reconstruction problems which have been shown to be highly applicable to respiratory‐resolved lung MRI [11, 15, 16]. Most notably, we demonstrate that these reconstructions are possible in clinically feasible timelines (< 10 min) with cheaper server‐grade (48 GB) GPUs, ultimately making respiratory‐resolved lung MRI more clinically accessible.

## 2 Theory

### 2.1 Objective Function

In this work, we target objective functions of the form:

(1) $$x^{*}=\text{argmi}n_{x}\frac{1}{2}{\left\|P^{\frac{1}{2}}\left(\mathrm{FSx}-k\right)\right\|}_{2}^{2}+g\left(x\right),$$

where $x\in {\mathbb{C}}^{R\times J}$ is the underlying image being solved with R respiratory phases and J voxels, $k\in {\mathbb{C}}^{R\times C\times K}$ is the acquired k‐space data with C coil channels and K measurements per respiratory phase; $P\in {\mathbb{C}}^{R\times K}$ is the k‐space preconditioner to assist with convergence [17]; $F\in {\mathbb{C}}^{R\times K\times J}$ is the Fourier encoding operator that maps complex images from each respiratory phase to a single point in k‐space (computationally, this is handled using a non‐uniform Fast Fourier Transform [18]); $S\in {\mathbb{C}}^{C\times J}$ is the sensitivity map; ${\left\|\cdot \right\|}_{2}^{2}$ denotes the $L_{2}$ norm which enforces data consistency; and g(x) is the regularization function.

### 2.2 Regularization

For respiratory‐resolved 4D lung MRI, a g(x) that enforces sparsity along the temporal dimension is desirable. A common choice is total variation (TV) regularization:

(2) $$g\left(x\right)=\lambda {\left\|\mathrm{Gx}\right\|}_{1},$$

where λ is the regularization strength parameter and G is the spatio‐temporal finite differences linear operator. TV regularization is powerful for preserving sharp edges (e.g., the diaphragm/lung boundary), but can also be prone to staircasing artifacts between respiratory phases and over‐smoothing of lung parenchyma if regularized too strongly [3, 19].

Another common choice of regularizer is low‐rank (LR) regularization [20]:

(3) $$g\left(x\right)=\lambda {\left\|x\right\|}_{*},$$

where ${\left\|x\right\|}_{*}$ denotes the nuclear norm of x. LR regularization is particularly effective for respiratory‐resolved lung MRI, as the rank of the image stack should be low due to inherent similarity across respiratory phases, which leads to accurate signal preservation in low signal regions like the lungs [16]. Recently, this approach was advanced to account for respiratory motion by applying low rank regularization after spatially aligning the respiratory phases to a reference state [15]:

(4) $$g\left(x\right)=\lambda {\left\|\mathrm{Mx}\right\|}_{*},$$

where M is the linear operator that registers a set of images to a single state (e.g., end expiration). This approach is denoted MoCo‐LR and has demonstrated better preservation of anatomical structure and respiratory motion when compared to standard LR or TV regularization, particularly for reconstructions with large numbers of respiratory bins and subsequent under‐sampling [15, 21].

### 2.3 Optimization Algorithms

For the regularization functions defined above, the optimization problems can be solved using proximal operator‐based algorithms [22]. These include proximal gradient descent [23], Fast Iterative Shrinkage‐Thresholding Algorithm (FISTA) [24], and primal‐dual methods such as Primal Dual Hybrid Gradient (PDHG) [25], all of which have been implemented into widely‐used MRI reconstruction software packages such as BART, SigPy, and Gadgetron [26, 27, 28].

Despite their flexibility, proximal algorithms can be computationally expensive—especially for high‐resolution 4D lung MRI—because each iteration requires one or more calculations of the gradient of the data consistency function, given by:

(5) $$\nabla f\left(x\right)=A^{H}\left(\mathrm{Ax}-y\right),$$

where $A=P^{\frac{1}{2}}\mathrm{FS}$ is the effective encoding matrix with preconditioning weights and coil sensitivity maps; and $y=P^{\frac{1}{2}}k$ are the preconditioned measurements. The primary computational bottleneck arises from the calculation of $A^{H}\mathrm{Ax}$, which can be prohibitively large for high‐resolution 4D lung MRI due to the high dimensionality of F and S. Consequently, both the GPU memory footprint and per‐iteration computational burden can be substantial, often leading to reconstruction times that exceed clinical feasibility (e.g., tens of minutes on > 60 GB server‐grade GPUs).

### 2.4 Coil Compression

To reduce the computational burden, coil compression is often used to project the original set of C coil measurements into a smaller set of $C^{\prime }\ll C$ virtual coils while retaining most of the signal energy [12]. A common approach is SVD‐based coil compression, which operates on the coil covariance matrix [13]. Following eigen‐decomposition, the highest energy eigenvectors form a compressed k‐space matrix occupying a considerably smaller volume than the original k‐space matrix and sensitivity maps. Subsequently, the data consistency term in Equation (1) can be reformulated and solved with reduced computational burden. However, this reduction in size may also lead to loss of coil‐specific information; inconsistent sensitivity profiles across the image; and increased noise sensitivity [13]. All of these can have profound impact on lung MRI, where fine details and parenchymal SNR are critical.

### 2.5 Coil Sketching

Coil sketching is a similar computational strategy to coil compression, but instead of applying a fixed linear projection on the coils, it iteratively reconstructs the image using randomly selected subsets of coils. At each outer iteration t, a sketched encoding operator, $A_{S}^{t}=P^{\frac{1}{2}}FS_{S}^{t}$ is formed from a random combination of $C_{S}^{t}\ll C$ coils, where $S_{S}^{t}\in {\mathbb{C}}^{C_{S}^{t}\times J}$ are the sketched sensitivity maps. The reconstruction then alternates between solving a reduced data consistency problem using $A_{S}^{t}$ in the inner loop, and using the full operator A to compute a guiding gradient once per outer loop:

(6) $$\nabla f_{S}^{t}\left(x\right)={A_{S}^{t}}^{H}A_{S}^{t}\left(x-x^{t}\right)+A^{H}\left(Ax^{t}-y\right).$$

This hybrid approach allows compatibility with standard proximal algorithms, as the gradient structure remains similar—differing only by an additional quadratic term. Importantly, the intermittent use of the full operator A ensures convergence toward the solution of the fully sampled system. Furthermore, because the $A^{H}A$ term is only evaluated on the outer loop, it can be batched into smaller subsets of coils to lighten the memory footprint and GPU load at low cost to total reconstruction duration. The result is significant acceleration and memory savings, without compromising reconstruction accuracy [14].

In this work, we expand the coil sketching algorithm, which was previously proposed for 3D data, to 4D (3D + time). The fundamental application of the algorithm remains unchanged, as the 4D data consistency term iterates over each respiratory phase separately, thus effectively solving a dynamic series of 3D problems. However, the primary advantages of coil sketching: reduced memory load and faster computation, are expected to have significant impact in high‐resolution lung MRI which suffers from prohibitively large matrix sizes and slow computation.

### 2.6 Toeplitz Approximation

Solving the data consistency term using iterative methods requires several $A^{H}A$ evaluations. As each $A^{H}A$ evaluation involves two NUFFT operations (forward and adjoint), this process can be slow when the number of k‐space measurements, K, is large, as the interpolation of non‐Cartesian measurements onto a Cartesian grid is computationally expensive. As described by Fessler et al. [29], this process can be accelerated by approximating the normal operator $A^{H}A$ as a Toeplitz operator, T:

(7) $$T\approx A^{H}A.$$

Toeplitz approximation converts the $A^{H}A$ operation, which requires computationally expensive k‐space interpolations, into an operation that computes entirely in Cartesian space (i.e., no k‐space interpolations are required before performing an FFT). As a result, it can be significantly faster than the standard $A^{H}A$ operation. Importantly, the Toeplitz point spread function depends only on the sampling trajectory in F, not on the coil sensitivities. As such, it is equally applicable to both the full operator A and the sketched operator $A_{S}^{t}$, allowing the computational benefits of Toeplitz acceleration to extend naturally to coil‐sketching reconstructions.

## 3 Methods

### 3.1 Human Participants and Scanner Details

Imaging was performed on healthy volunteers and patients with local IRB approval (clinicaltrials.gov NCT03331380) and all subjects provided written informed consent. In this study, imaging data were acquired in 5 healthy volunteers and 5 patients with LAM. Images were acquired on a 0.55 T MRI scanner (prototype MAGNETOM Free.Max, Siemens Healthineers AG, Forchheim, Germany) with a chest and spine coil comprising a total of 15 coil‐elements.

### 3.2 Acquisition Parameters

Data were acquired using a free‐breathing 3D stack‐of‐spirals VIBE ultrashort echo time sequence (Siemens Healthineers AG, Erlangen, Germany). Sequence parameters included: TE/TR = 0.70/8.3 ms; FA = 8°; FOV = 480 × 480 × 190 mm^3^; matrix size = (288 × 288 × 112) with an additional 14.3% oversampling in z; readout duration = 5.4 ms; dwell time = 2 μs; scan‐duration = 8:29 min:s; coronal acquisition; golden angle ordering; 397 spiral interleaves per k_z_. RF excitations were performed using a slab‐selective pulse covering the full 190 mm superior–inferior block of the prescribed FOV. Respiratory motion was resolved with a superior–inferior navigator readout acquired every 120 ms [30]. Trajectories were retrospectively corrected for gradient errors using the gradient impulse response function [31, 32].

### 3.3 Reconstruction Parameters and Pipeline

**FIGURE 1:**
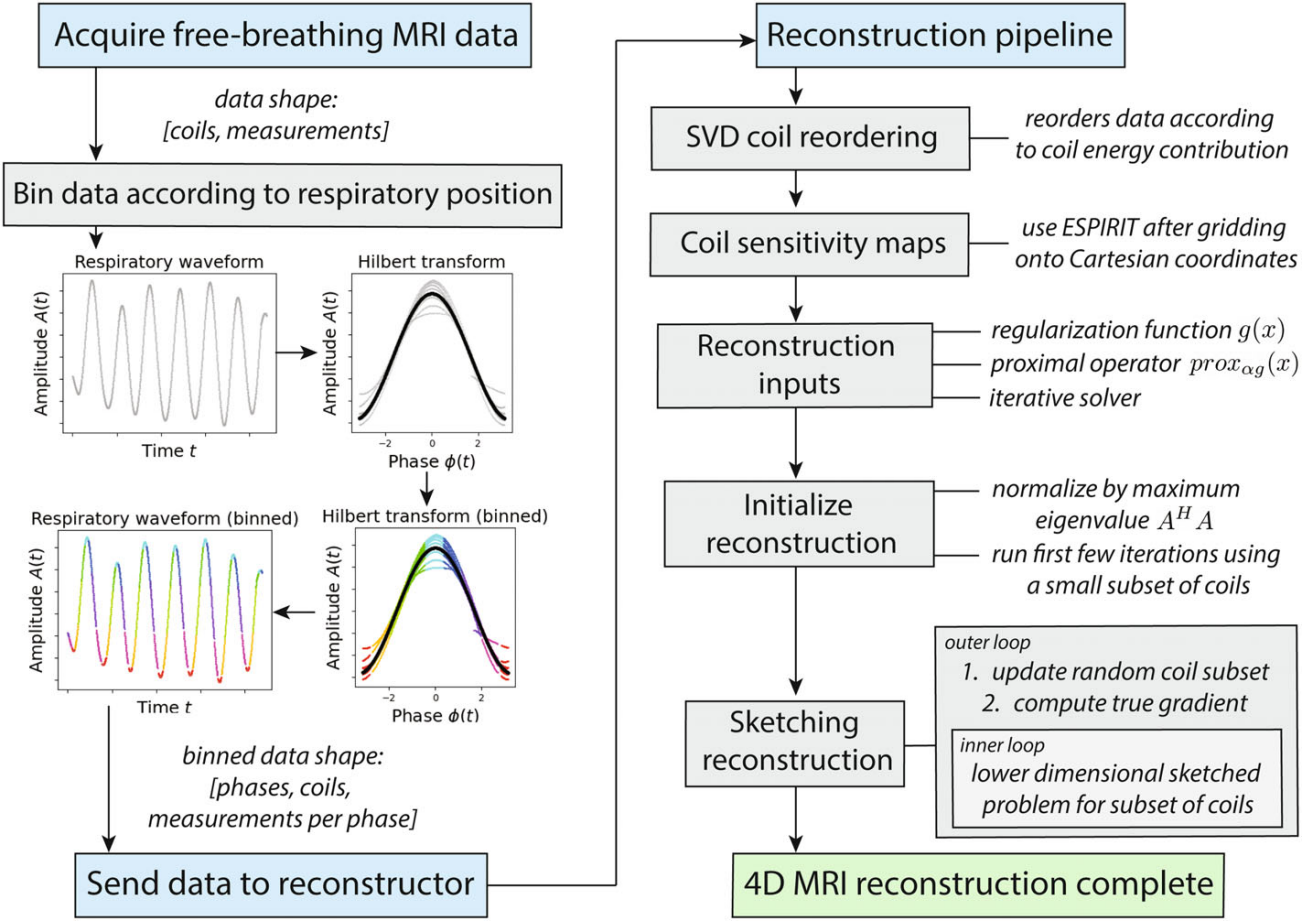
Respiratory‐resolved lung MRI coil‐sketched reconstruction pipeline. Starting from data acquisition, raw data are binned according to respiratory position and sent to the reconstructor. The *sketching reconstruction* module can be replaced with the coil compressed or conventional reconstruction modules.

Reconstructions were performed using SigPy [26] toolbox in Python 3.10 on a single NVIDIA‐A100 80 GB GPU (Nvidia Corporation, Santa Clara, CA). A flowchart for the reconstruction pipeline is shown in Figure 1. Firstly, the waveform acquired using the superior–inferior navigator [30] was binned into R respiratory states according to the phase (obtained by a Hilbert Transform) in the respiratory cycle, as previously described [33]. The indices of each measurement were used to bin the raw k‐space data and coordinates into respiratory states, resulting in a data structure with K measurements per respiratory state.

Secondly, singular value decomposition (SVD) was used to reorder k‐space data according to coil energy contribution, such that contributions for each coil were ranked from highest in the first virtual coil, to lowest in the final virtual coil. Next, coil sensitivity maps were estimated using the ESPIRIT algorithm [34] after gridding the non‐Cartesian measurements onto a 2‐fold downscaled Cartesian grid and then upscaling back to the full matrix size. Next, k‐space preconditioning weights were calculated using the single‐channel non‐Cartesian k‐space preconditioner method [17]. Finally, the maximum eigenvalue of the normal operator $A^{H}A$ was estimated using the Power Method and used to normalize A such that its eigenvalue spectrum ranged from approximately 0 to 1 (important for stable convergence and for robustness) [24].

For each reconstruction, the following initialization inputs were chosen prior to reconstructing: regularization function g(x); corresponding proximal operator function ${\text{prox}}_{g}\left(x\right)$; and a suitable iterative solver. Unless stated otherwise, all *sketching* reconstructions used 5 initial iterations, 5 inner iterations per sketched sub‐problem, and 6 outer iterations (i.e., 6 different sketched coil combinations). Initial iterations comprised an SVD coil compressed reconstruction, where reconstructions were performed using the $C_{S}$ coils with the highest energy. Subsequent sketched reconstructions were performed using random sketched coil subsets (comprised of $C_{S}^{t}$ coils), where 1 coil was maintained across all iterations and $\left(C_{S}^{t}-1\right)$ sketched coils were randomly selected for each outer iteration t. Random coil selection was performed using a Rademacher distribution. All reconstructions used the same total number of iterations. Hyperparameter λ was set to 0.1 for all reconstructions. The choice of regularization and specific reconstruction settings are given in the next subsection. Finally, all images were reconstructed onto a 1.33‐times larger matrix to prevent wrap‐in artifacts caused by signal outside the reconstructed FOV.

### 3.4 Motion‐Compensated Low Rank Regularization Implementation

To implement the MoCo‐LR cost function (Equation 4), motion fields *M* were estimated using a GPU‐accelerated optical flow algorithm in Python [35]. These fields were non‐diffeomorphic—that is, forward and inverse transformations were not exactly invertible. Consequently, operations that involve successive forward and inverse transformations could result in artifacts consistent with poor registrations [36]. Inconveniently, this makes the MoCo‐LR implementation described by Tan et al. [15] challenging, as the proximal update for ${\left\|\mathrm{Mx}\right\|}_{*}$ involves numerous forward‐inverse transformations, and can lead to iterative instability or artifact amplification.

To address this, we designed a novel proximal update implementation that only involves forward transformations. At each outer iteration, one respiratory phase was selected as the reference. All other phases were registered to this reference to align the stack. Low‐rank soft‐thresholding was then applied along the respiratory dimension, and only the updated reference frame was retained. This process was repeated across all respiratory phases, ensuring that each frame was updated in a motion‐compensated low‐rank framework without requiring backward deformation.

An algorithm for the forward‐only proximal operator update is given in Algorithm 1.

ALGORITHM 1 **Motion‐compensated low rank proximal operator update.**

**Initialize:**

$x_{\text{in}}$ = input 4D image matrix

$x_{\text{out}}$ = output 4D image matrix

**For** *reference phase* **in** *total respiratory phases*:

  

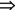
 Register all phases of $x_{\text{in}}$ to *reference phase*

  

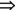
 Reshape registered 4D image stack to R respiratory states × J voxels

  

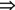
 Perform SVD on reshaped image stack

  

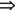
 Soft‐threshold singular values

  

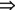
 Reconstruct image stack and reshape back to 4D

  

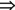
 Store only the *reference phase* index of the regularized image stack in $x_{\text{out}}$

### 3.5 Experiments Performed

#### 3.5.1 Experiment 1

Goal: demonstrate the respiratory‐resolved sketching reconstruction pipeline using common regularization functions: (i) ${\left\|x\right\|}_{2}^{2}$ (CG‐SENSE); (ii) ${\left\|\mathrm{Gx}\right\|}_{1}$ (spatial–temporal TV); (iii) ${\left\|x\right\|}_{*}$ (LR reconstruction); and (iv) ${\left\|\mathrm{Mx}\right\|}_{*}$ (MoCo‐LR reconstruction). Conjugate‐gradient descent was used as the solver for CG‐SENSE and PDHG (with 4 inner iterations for the primal update) was chosen for all other reconstructions.

This experiment was performed on a dataset acquired in a patient with LAM and reconstructed using 12 respiratory states.

#### 3.5.2 Experiment 2

Goal: compare reconstructed image accuracy using a varying number of virtual coils out of the total (15) available coils with SVD coil compressed reconstruction versus the proposed coil sketching reconstruction. Image error was assessed using the normalized root mean square error of the lung region (NRMSE) comparing the images with reduced virtual channels to images with no coil compression applied. Additionally, measure the maximum GPU memory footprint during the reconstruction.

This experiment was performed over the entire study population (*N* = 10) using 6 respiratory states for the reconstruction. Images were reconstructed using the MoCo‐LR approach, using PDHG as the solver.

#### 3.5.3 Experiment 3

Goal: examine convergence curves for the same reconstruction in Experiment 2 between: (i) conventional reconstruction with 15 virtual coils; (ii) conventional reconstruction with 15 virtual coils, iterating through coil batches containing 3/15 virtual coils each; (iii) compressed coil reconstruction using the 3 virtual coils with the highest energy; (iv) coil sketched reconstruction using 3 total sketched coils. Convergence was assessed by dividing the value of the objective function at each iteration relative to the value of the objective function after 1000 total iterations (5 initial; 5 inner; 199 outer iterations for the sketched formulation).

#### 3.5.4 Experiment 4

Goal: compare reconstructions using Toeplitz approximation inside the gradient step update. Reconstruction accuracy was assessed using the difference between the standard and Toeplitz methods, for both conventional and coil sketching (3 sketched coils) reconstruction. Difference was assessed using NRMSE. Additionally, convergence speed was assessed between each method to see if reconstruction speed gains were possible.

This experiment was performed using MoCo‐LR with PDHG as the solver and 6 respiratory states on a healthy volunteer dataset.

#### 3.5.5 Experiment 5

Goal: compare maximum GPU memory load and iteration speed for MoCo‐LR and LR reconstructions for a range of respiratory bins (2–20), using conventional and sketched (3 sketched coils) reconstructions, with and without Toeplitz acceleration.

#### 3.5.6 Experiment 6

Goal: compare the SNR between the conventional, coil compressed, and coil sketched reconstructions (with and without Toeplitz approximation). SNR was measured using the pseudo‐replica approach [37], with steps as follows: (i) pre‐whiten raw data such that the standard deviation of the noise was equal to 1; (ii) add random complex Gaussian noise (standard deviation = 1) to raw data; (iii) reconstruct image; (iv) repeat steps (i–iii) 30 times (i.e., 30 pseudo‐replicas); (v) measure SNR using the mean signal divided by the standard deviation signal for each voxel, measured along the repeated pseudo‐replica dimension.

## 4 Results

### 4.1 Experiment 1: Regularization Comparison

**FIGURE 2:**
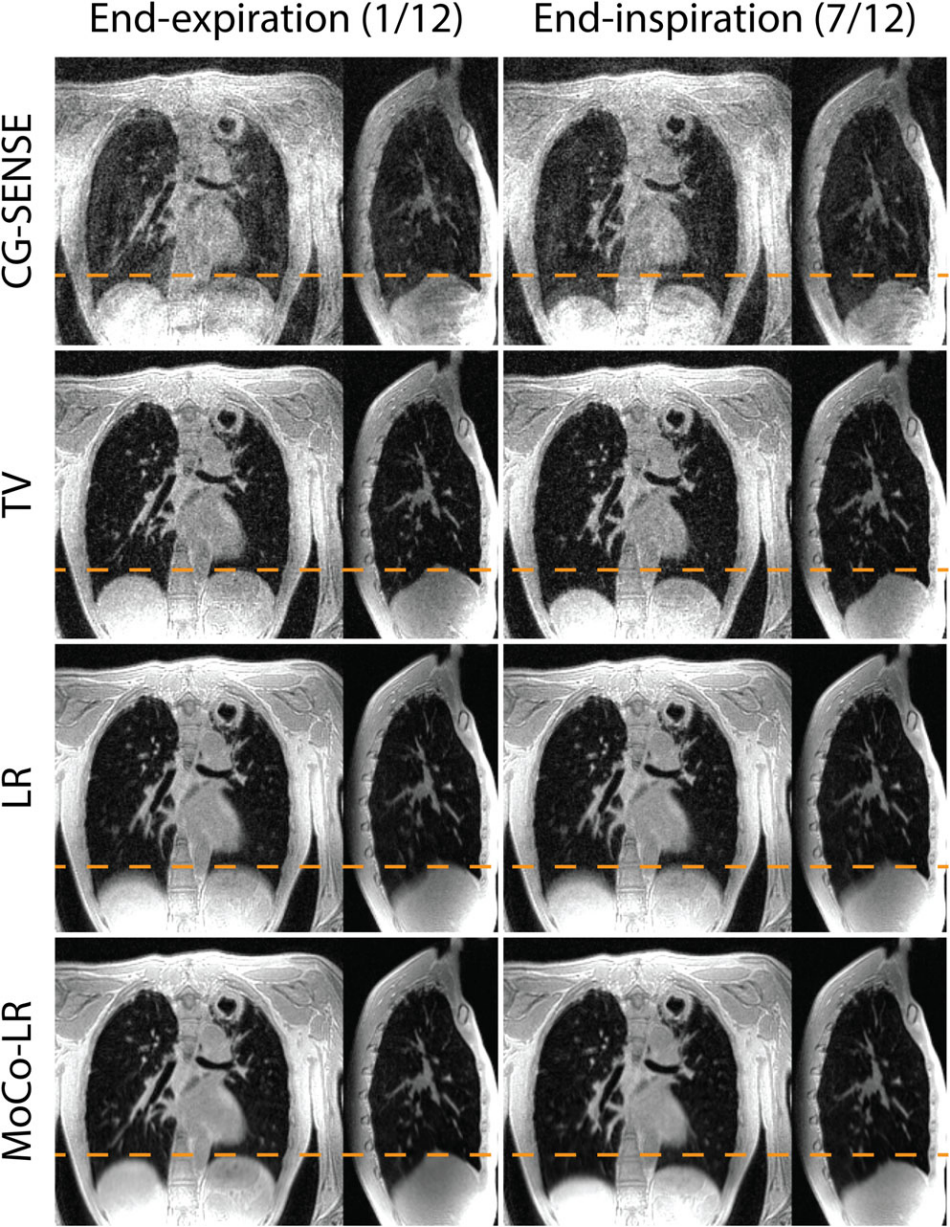
Comparison of 4D coil sketching reconstructions using different regularizations (CG‐SENSE, spatio‐temporal TV, LR, and MoCo‐LR) at two different respiratory states among the 12. The orange dash line represents the diagram position at end‐expiration state. All reconstructions used λ = 0.1, however, an additional parameter sweep of λ is given in the Supporting Figure S1.

In Figure 2, coil sketching was used to successfully reconstruct respiratory‐resolved lung MRI data with 12 respiratory bins, using CG‐SENSE; spatio‐temporal TV; LR; and MoCo‐LR reconstruction approaches, demonstrating the versatility of the coil sketching approach at solving 4D MRI problems. Source code for each of these reconstruction approaches has been made available open‐source here: https://github.com/NHLBI/lit_python_toobox/tree/coil‐sketching‐4d. Reconstruction times were 2:21, 5:14, 5:14, and 9:08 min, respectively. These times do not include the times to estimate the coil sensitivity maps and k‐space preconditioner, which were 51 s total and consumed < 20 GB of memory. An additional parameter sweep of λ for each reconstruction approach is performed in Figure S1. From this point, all further experiments used the MoCo‐LR reconstruction approach with λ = 0.1.

### 4.2 Experiment 2: Coil Compressed Versus Coil Sketching Reconstruction

**FIGURE 3:**
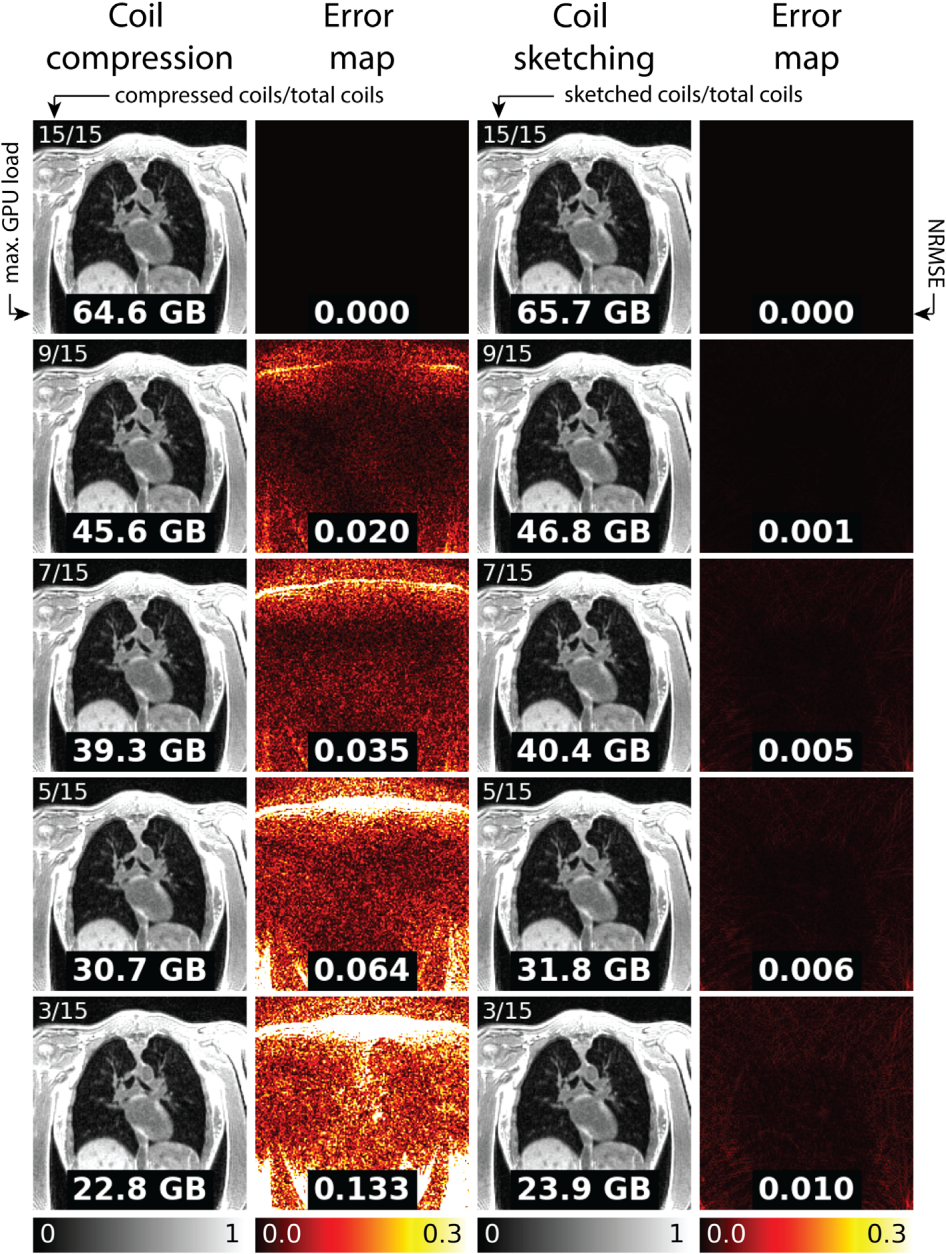
Comparison of memory footprint and accuracy between SVD coil compression and coil sketching reconstructions, with 3–15 coils, for 4D lung MRI (binned into 6 respiratory phases) using MoCo‐LR reconstruction. Each image has a maximum GPU memory allocation overlaid. Error maps are included with a normalized root mean square error (NRMSE) measurement overlaid.

**FIGURE 4:**
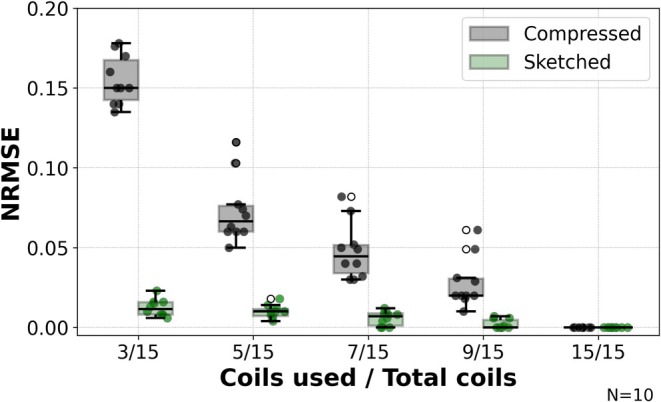
Comparison of NRMSE measurements in 10 subjects between a coil compressed reconstruction and coil sketching reconstruction with 3–15 virtual or sketched coils, respectively across the study population using the same settings as Figure 3. Reference measurement is the conventional reconstruction without any coil compression.

In Figure 3, example 4D MoCo‐LR lung images (binned into 6 respiratory phases) from a single healthy volunteer are provided showing a range of virtual or sketched channels (3 to 15). Both SVD coil compressed and coil sketching reconstructions reduced maximum GPU memory footprint from the fully sampled conventional reconstruction by nearly 3‐fold when using only 3/15 coils (˜40 GB memory reduction). For SVD coil compression, the image error increased with further compression, with a maximum NRMSE of 0.133 when using only 3 virtual coils. The coil sketching reconstruction retained better accuracy, with maximum NRMSE of 0.010 for 3 sketched coils. In Figure 4, NRMSE across 5 healthy volunteers and 5 patients with lymphangioleiomyomatosis for 3/15 virtual coils was 0.150 ± 0.023 for SVD coil compression, and 0.010 ± 0.004 for coil sketching, further demonstrating the retained accuracy at very few channels. Three virtual coils were used for the remaining analyses.

### 4.3 Experiment 3: Convergence Analysis

**FIGURE 5:**
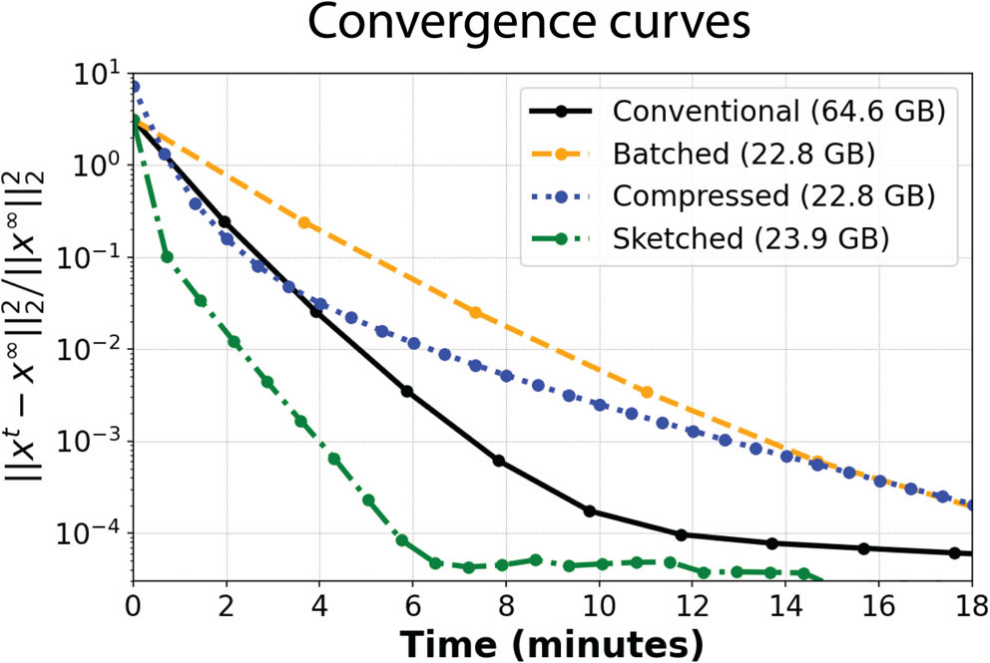
Convergence curves for the conventional, coil batched (with 3 coils per batch), coil compressed (3/15 coils), and coil sketched (3/15 sketched coils), for the dataset demonstrated in Figure 3. Dotted markers indicate the time stamp for every fifth iteration.

Figure 5 contains convergence curves for a 4D MoCo‐LR 6‐phase reconstruction using: conventional reconstruction without compression (15/15 coils); batched reconstruction (15/15 coils, with 3 coils per batch); coil compressed reconstruction (3/15 virtual coils); and sketched reconstruction (3/15 sketched virtual coils). Of these, the coil sketched reconstruction showed the fastest convergence and utilized > 60% less memory than the conventional reconstruction. The batched reconstructions and coil compressed reconstructions used less memory, however, their convergence was notably slower. As an example: when using 20 total iterations, the reconstruction times were 7:53, 14:20, 2:19, and 2:28 mm:ss for the conventional, batched, coil‐compressed, and coil‐sketched reconstructions, respectively.

### 4.4 Experiment 4: Toeplitz Approximation of the Normal Operator

**FIGURE 6:**
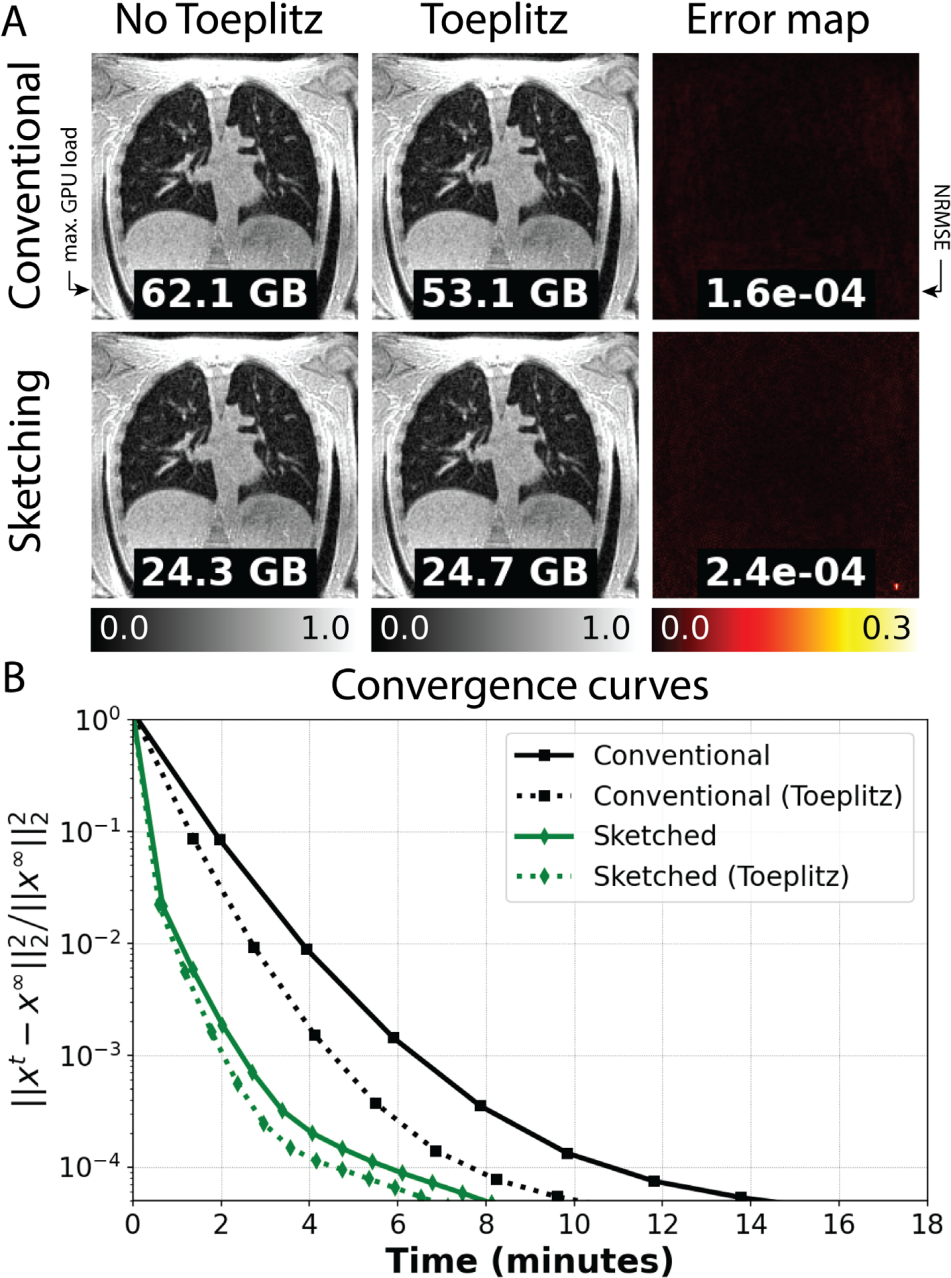
Comparison of (A) memory footprint, precision using NMRSE and (B) convergences curves between conventional and coil sketched with and without Toeplitz. The coil sketched reconstruction was performed using 3/15 coils. Dotted markers indicate the time stamp for every fifth iteration.

In Figure 6A, image reconstructions with and without Toeplitz approximation of the $A^{H}A$ normal operator are shown for conventional and sketched reconstructions. Qualitatively, the error maps between Toeplitz/non‐Toeplitz show that the impact of Toeplitz approximation is small (NRMSE ˜10^−4^). In this example, the additional memory load caused by storing the Toeplitz linear operator on the GPU is reflected in the slight memory (0.4 GB) increase in the sketched Toeplitz versus non‐Toeplitz reconstructions, however, the conventional reconstruction experiences a memory saving (9.0 GB) when using Toeplitz.

In Figure 6B, the convergence curves for the reconstructions in (A) are shown, where the sketched reconstruction using Toeplitz approximation shows the fastest convergence (approximately 3‐fold faster than a conventional reconstruction), such that reconstruction time for 20 iterations was reduced to 2:15 min.

### 4.5 Experiment 5: Reconstruction Efficiency for Varying Number of Respiratory States

**FIGURE 7:**
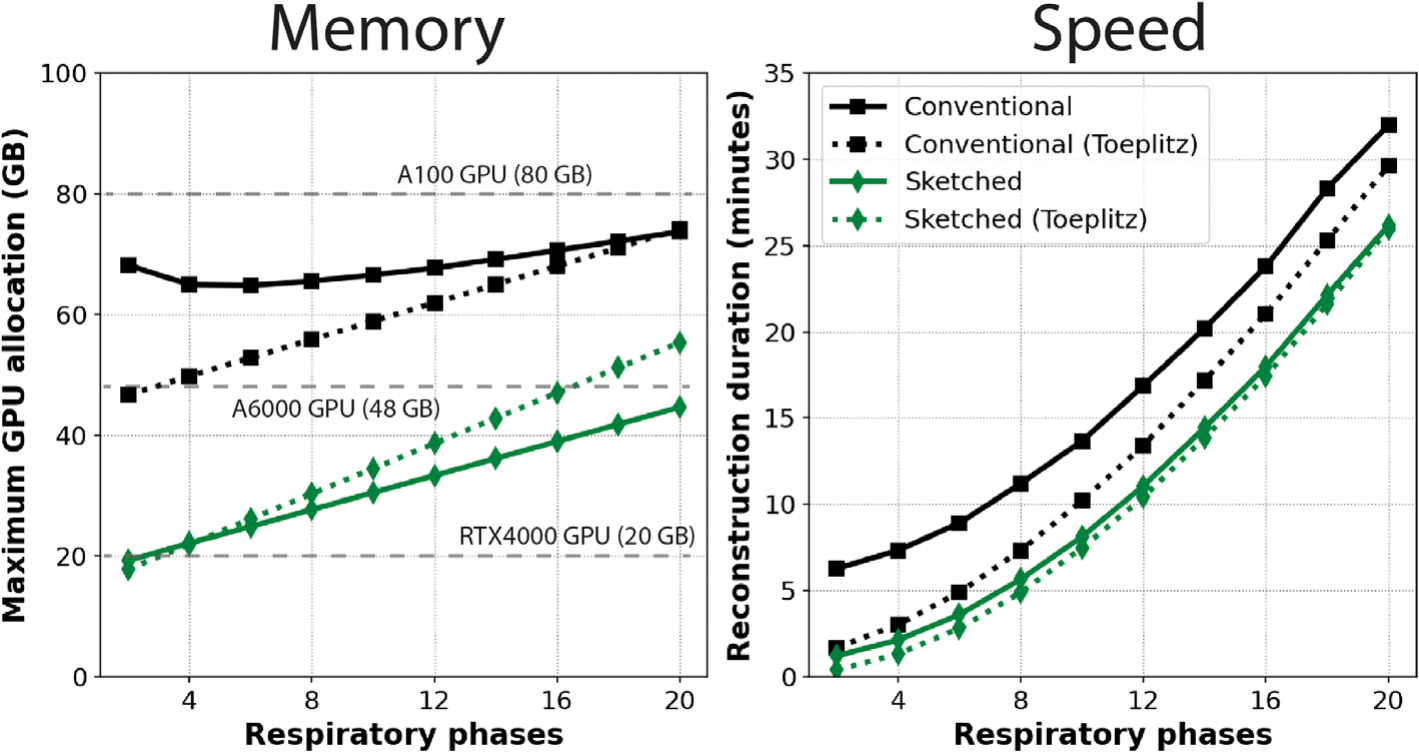
Memory consumption and iteration speed for conventional, coil sketched (3/15 sketched coils), with and without Toeplitz approximation, for MoCo‐LR reconstructions with 2–20 respiratory phases. Commercially available NVIDIA GPUs (A100; A6000; and RTX4000) are shown as examples on the memory plot.

In Figure 7, the maximum memory load on the GPU and the reconstruction speed (iteration duration) are shown for a varying number of respiratory states (2 to 20). Notable observations include: (i) the maximum memory used by the sketching reconstructions is lower than that for the conventional reconstructions, in this case, fitting onto substantially cheaper GPUs (< 48 GB); (ii) the maximum memory consumption increases with the lower number of respiratory phases for the sketching and conventional reconstructions, due to the large volume of data in each respiratory bin when fewer phases are used; (iii) the use of Toeplitz approximation saves significant memory for the conventional reconstruction but increases memory load for the sketched reconstruction for larger numbers of respiratory phases; (iv) the reconstruction speed is faster for the Toeplitz sketched reconstruction, particularly when fewer respiratory phases are used; and (v) reconstruction duration monotonically increases with increasing respiratory phases. In Supporting Figure S2, the same experiment is performed, but with LR reconstruction instead of MoCo‐LR to exclude the impact of motion compensation. There, similar trends were observed with a more a linear relationship between speed and respiratory phases.

### 4.6 Experiment 6: SNR For Each Reconstruction Approach

**FIGURE 8:**
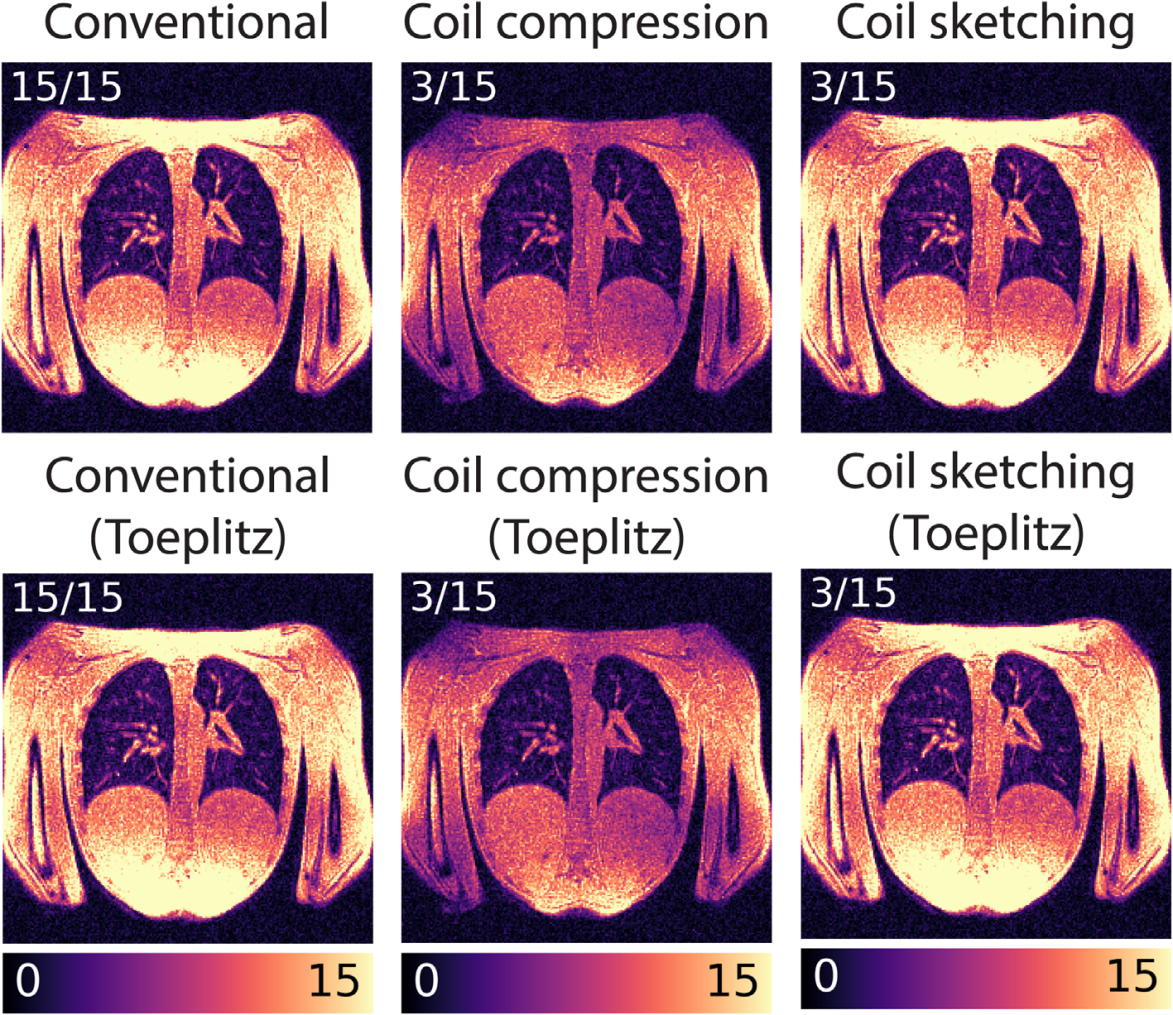
Comparison of SNR maps for the conventional, coil compressed, and coil sketched reconstructions, with and without Toeplitz approximation.

In Figure 8, pseudo‐replica SNR maps are shown for the conventional, coil compressed, and coil sketched reconstructions, with and without Toeplitz. Qualitatively, all approaches show similar SNR variations across space, except for the coil compressed reconstruction, which exhibits regions of low SNR across the upper/lower torso.

### 4.7 Demonstration in Volunteers and Patients

**FIGURE 9:**
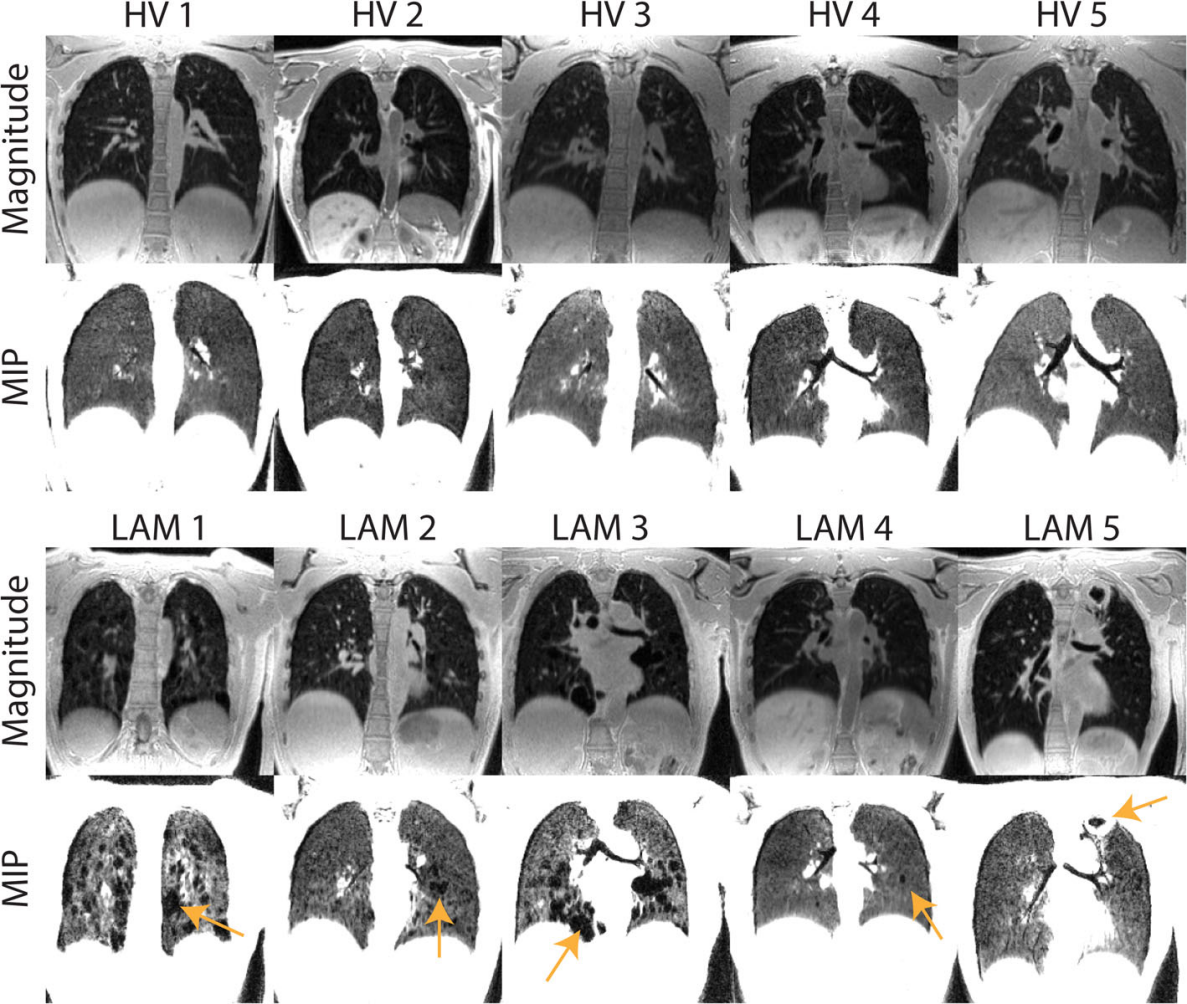
Single slice examples across five healthy volunteers (HV) and five patients with lymphangioleiomyomatosis (LAM) using coil sketching with Toeplitz (MoCo‐LR reconstruction with 12 respiratory phases). Minimum intensity projections across the 10 adjacent coronal slices are also shown to help delineate cysts. Orange arrows point to notable cysts in patients with LAM.

In Figure 9, a single slice at end‐inspiration is shown across our study population of 5 healthy volunteers and 5 patients with LAM using coil sketching with Toeplitz (MoCo‐LR reconstruction with 12 respiratory phases). Minimum intensity projections across the 10 adjacent coronal slices are also shown to help delineate cysts. Across different body habitus, high quality imaging was possible, and clear cyst delineation is visible in patients with LAM. Each reconstruction was terminated at 8:29 min (the same duration as the acquisition), and the maximum memory consumption was 36.0 GB.

## 5 Discussion

We present several novel features that advance respiratory‐resolved lung MRI reconstruction. Firstly, we extend the coil sketching framework, originally proposed for 3D MRI [14], to 4D imaging. Critically, this helps solve the unique computation challenges associated with very‐large matrix sizes and respiratory motion for 4D lung MRI. Secondly, we employ the coil sketching framework to efficiently solve MoCo‐LR reconstruction problems that have been previously reported to work particularly well for respiratory‐resolved lung MRI [11, 15, 21]. Finally, we apply Toeplitz approximation to accelerate the normal operator $A^{H}A$, replacing the computationally expensive NUFFT operations with faster FFTs, benefitting both the conventional and sketched reconstructions. Notably, these reconstructions take < 48 GB of GPU consumption and reconstruct on the order of < 10 min, while previous reports took the order of hours [15].

The key principle behind coil sketching is that the data consistency term in the conventional reconstruction is broken down into smaller subproblems, each comprised of a minimization task using a random “sketched” subset of coils and an additional gradient term to help guide convergence. This is similar to a coil compressed reconstruction where only a fraction of coils is considered, however, instead of converging within the compressed coil space, sketching converges within the fully sampled coil space used by conventional reconstruction. This is important, as memory consumption and computation speed are significantly reduced with coil sketching, while image accuracy is preserved compared to standard coil compression. Notably, these gains are made using adjustments that only target the data consistency term, while the regularization term and iterative procedure remain the same. Consequently, coil sketching is highly generalizable to various MRI reconstruction problems. This generalizability is demonstrated in Figure 2, where 4D coil sketching reconstructions are shown for CG‐SENSE, TV, LR, and MoCo‐LR.

In this work, we focused on the MoCo‐LR reconstruction due to its proven efficacy for respiratory‐resolved lung imaging [11, 15, 21]. However, in comparison to the original works by Tan et al. [15], we modified the soft‐thresholding procedure such that the proximal operator for the motion‐compensated images only involves a forward operation (i.e., no inverse registration was required) since the linear operator that applies motion fields M is not strictly diffeomorphic. This improves stability of the reconstruction as it no longer uses iterative forward/inverse deformations, which grow unstable over larger numbers of iterations [36]. While this method increases the total number of registrations by a factor of R respiratory states, the process remains fast on a GPU, with each registration completing in under 0.2 s. However, we recognize that this efficiency may be less applicable for larger matrices (higher resolution). Future work could improve this further by incorporating GPU‐based diffeomorphic registration toolboxes like FireANTs [38].

The computational cost, image accuracy, and convergence speed are well elucidated in Figures 3, 4, 5. In Figure 3, the memory consumption decreases with fewer coils, as expected, for both SVD coil compression and coil sketching reconstructions. However, it is noteworthy that the loss of signal and image accuracy caused by using fewer coils with the coil compressed reconstruction is largely mitigated using coil sketching. The coil sketching reconstruction consumes approximately 1.1 GB more memory for each experiment, due to the storage of the full‐gradient term in Equation (6) that must be added for each gradient step update. However, this additional memory consumption is small compared to the savings from using fully sampled conventional reconstructions.

In addition to coil sketching, our results indicate that Toeplitz approximation provides a significant convergence speedup with negligible loss of image accuracy. However, its use involves a memory trade‐off. The Toeplitz point spread function must be stored on the GPU, which slightly increased the total memory footprint of our sketched reconstructions by 0.4 GB. Conversely, for conventional, fully‐sampled reconstructions, the Toeplitz method saved 9.0 GB of total memory, as it reduced the number of memory‐intensive non‐Cartesian $A^{H}A$ operations. Overall, the benefits of Toeplitz approximation for speed are significant, however, the memory implications will need to be considered on a case‐specific basis, as they will depend on the number of coils and size of the reconstruction problem. One theoretical disadvantage of Toeplitz approximation is applicability to extremely‐large matrices, as the size of the Toeplitz point spread function increases 2‐fold with the image size (a total of 8‐fold in 3D), which may render some high‐resolution applications unfeasible [29]. However, the Toeplitz operation can be parallelized onto an additional GPU device if available.

For practical deployment, users may ask questions such as: how many respiratory phases can be reconstructed with MoCo‐LR on a 48 GB GPU? This is difficult to predict a priori, as maximum memory consumption depend on solver, regularization, matrix size, number of measurements, buffer usage, gridding parameters, and more. To explore this, we varied the number of respiratory phases and measured memory consumption and reconstruction speed (Figure 7). Notably, we found that reconstructions with fewer bins consumed more memory, likely because each phase contained more k‐space measurements, making the NUFFT operation more intensive. As the number of bins increased, memory consumption increased almost linearly, reflecting the larger 4D image volume. Reconstruction was consistently fastest for the Toeplitz‐sketched approach, notable given its memory advantages over conventional reconstruction. Runtime, however, grew quadratically with more bins, likely due to the increasing number of registration operations within the MoCo‐LR proximal update. This interpretation is supported by the more‐linear trends in Figure S2, where no motion compensation is performed. Combined with Figures 2, 3, 4, 5, 6, these findings provide practical guidance for integrating coil sketching into reconstruction pipelines.

There are limitations to the experiments performed in this manuscript. Firstly, we did not experiment with iterative solver optimizations. For example, the MoCo‐LR reconstruction can be solved using both FISTA and PDHG (both available in the code repository: https://github.com/NHLBI/lit_python_toobox/tree/coil‐sketching‐4d). Future work could explore these algorithm‐specific optimizations, in addition to comparisons with other stochastic gradient descent methods [16]. An additional limitation of this research is that we did not test in a wide range of non‐Cartesian sequences, such as bSTAR; spiral cones; or spiral FLORET [39, 40, 41, 42]. Nonetheless, the improvements demonstrated with coil sketching in 3D stack‐of‐spirals should generalize, as the method targets only the coil component of the encoding matrix. Our experiments also emphasized MoCo‐LR; additional speedups may be possible with TV or LR regularization, which could be attractive for certain applications.

Future work should explore these directions, as well as conceptual extensions to the sketching algorithm itself, such as sketching across temporal phases or optimizing sketching matrices (this work used the Hessian methods from the original publication [14]). Applying coil sketching to larger coil arrays or 5D cardio‐pulmonary reconstructions may also provide substantial memory savings.

Altogether, our proposed 4D coil sketching reconstruction has notable impact for lung imaging. By using coil sketching, one can reduce the size of the coil dimension (5‐fold demonstrated here), while enabling high quality, accurate, MoCo‐LR reconstructions, with more reasonable computational memory demands and reconstruction timelines. For many clinics, GPU availability and reconstruction time are the largest barriers to respiratory‐resolved lung MRI, which coil sketching directly addresses. Furthermore, the time gains made using sketching and Toeplitz could make inline reconstructions more feasible. In the future, this could be highly beneficial for applications including PREFUL MRI [43] or cardio‐pulmonary‐binned MRI that require high temporal resolution [44].

## 6 Conclusion

We present a 4D coil sketching framework that accelerates respiratory‐resolved lung MRI reconstruction by over 3‐fold and reduces GPU memory demands by more than 2‐fold, enabling motion‐compensated low‐rank reconstructions in under 10 min on medium‐sized GPU hardware. This approach may help improve the clinical feasibility of techniques such as Fourier decomposition or PREFUL by addressing computational challenges of large matrix sizes and motion. Future optimizations and broader testing could further advance its utility in pulmonary MRI.

## Conflicts of Interest

This research was supported by the Intramural Research Program of the National Institutes of Health (NIH). The contributions of the NIH authors are considered Works of the United States Government. The findings and conclusions presented in this paper are those of the authors and do not necessarily reflect the views of the NIH or the U.S. Department of Health and Human Services. The authors are investigators on a US Government Cooperative Research and Development Agreement (CRADA) with Siemens Healthcare, which includes the development of 0.55 T MRI.

## Supporting information


**Figure S1:** Parameter sweep for λ for the CG‐SENSE, spatio‐temporal TV, LR, and MoCo‐LR reconstructions shown in Figure 2. The panel to the right of each coronal image shows the kymograph consisting of a lung/diaphragm cross section drawn at each of the 12 respiratory phases.
**Figure S2:** Memory consumption and iteration speed for conventional, coil sketched (3/15 sketched coils), with and without Toeplitz approximation, for LR reconstructions with 2–20 respiratory phases. Identical settings to the MoCo‐LR implementation in Figure 7 are used, but without motion compensation. Commercially available NVIDIA GPUs (A100; A6000; and RTX4000) are shown as examples on the memory plot.

## Data Availability

Python scripts and sample data for the reconstruction algorithms (coil‐sketching‐4d) are available at https://github.com/NHLBI/lit_python_toobox/tree/coil‐sketching‐4d. The authors kindly encourage users to ask questions, post issues, and collaborate with us on this repository.
