# Supplementary material for: Coil Sketching for Fast and Efficient 4D Lung MRI Reconstruction
Source: Magn Reson Med. 2025 Nov 7;95(4):2241–53. doi: 10.1002/mrm.70169 (PMC12850557; doi:10.1002/mrm.70169)
Supplement: Supplementary file 1 — Figure S1: Parameter sweep for λ for the CG‐SENSE, spatio‐temporal TV, LR, and MoCo‐LR reconstructions shown in Figure 2. The panel to the right of each coronal image shows the kymograph consisting of a lung/diaphragm cross section drawn at each of the 12 respiratory phases. Figure S2: Memory consumption and iteration speed for conventional, coil sketched (3/15 sketched coils), with and without Toeplitz approximation, for LR reconstructions with 2–20 respiratory phases. Identical settings to the MoCo‐LR implementation in Figure 7 are used, but without motion compensation. Commercially available NVIDIA GPUs (A100; A6000; and RTX4000) are shown as examples on the memory plot. [file MRM-95-2241-s001.pdf]

Supporting Information

Coil sketching for fast and efficient 4D lung MRI reconstruction

Joseph W. Plummer[1], Pierre Daudé[1], Anastasia Tsakirellis[1], Jordan Taylor[1], Joel Moss[2], Rajiv Ramasawmy[1], Ahsan Javed[1], and Adrienne E. Campbell–Washburn[1,*]

[1]Cardiovascular Branch, Division of Intramural Research, National Heart, Lung, and Blood Institute, National Institutes of Health, Bethesda, MD, United States
[2]Critical Care Medicine and Pulmonary Branch, Division of Intramural Research, National Heart, Lung, and Blood Institute, National Institutes of Health, Bethesda, MD, United States
[*]Corresponding author.

This PDF contains Supporting Figures 1 and 2.

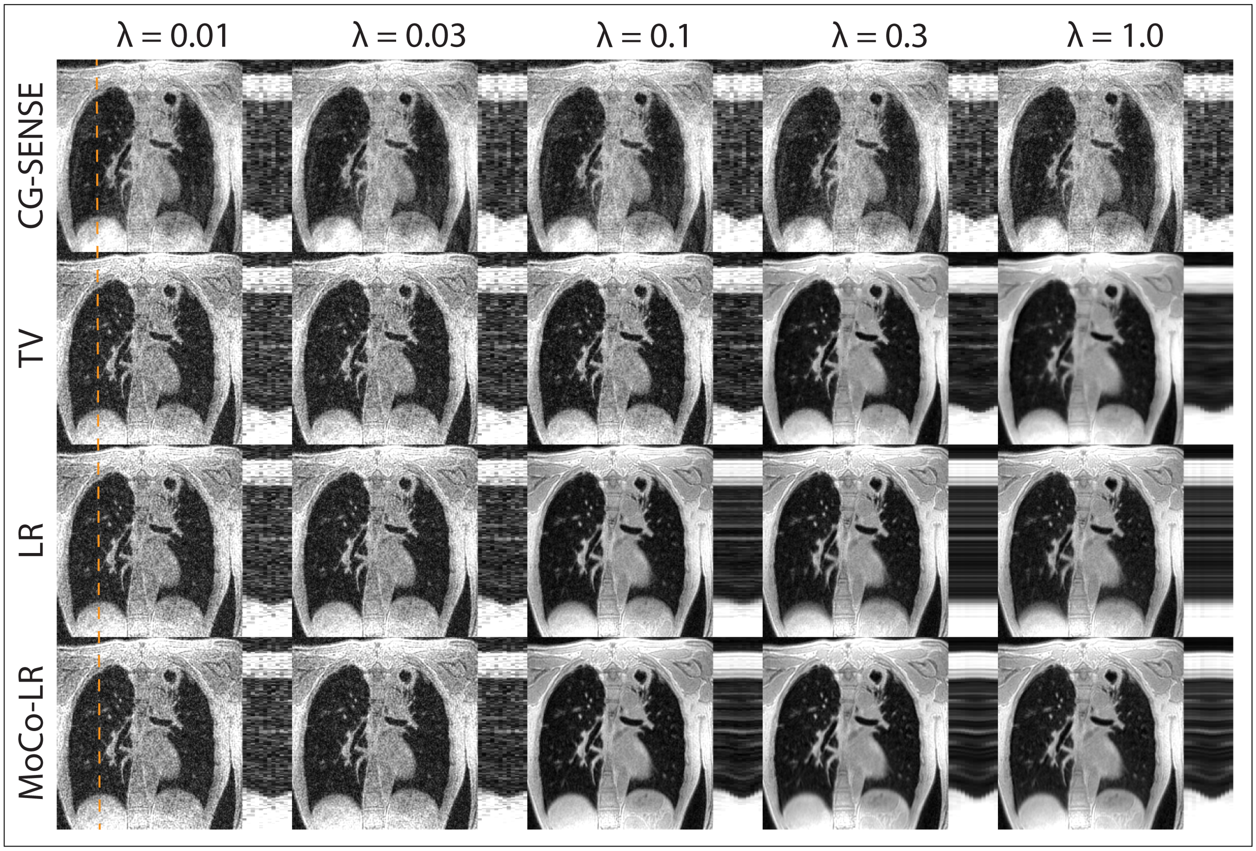

Supporting Figure 1: Parameter sweep for λ for the CG-SENSE, spatio-temporal TV, LR, and MoCo-LR reconstructions shown in Figure 2. The panel to the right of each coronal image shows the kymograph consisting of a lung/diaphragm cross section drawn at each of the 12 respiratory phases.

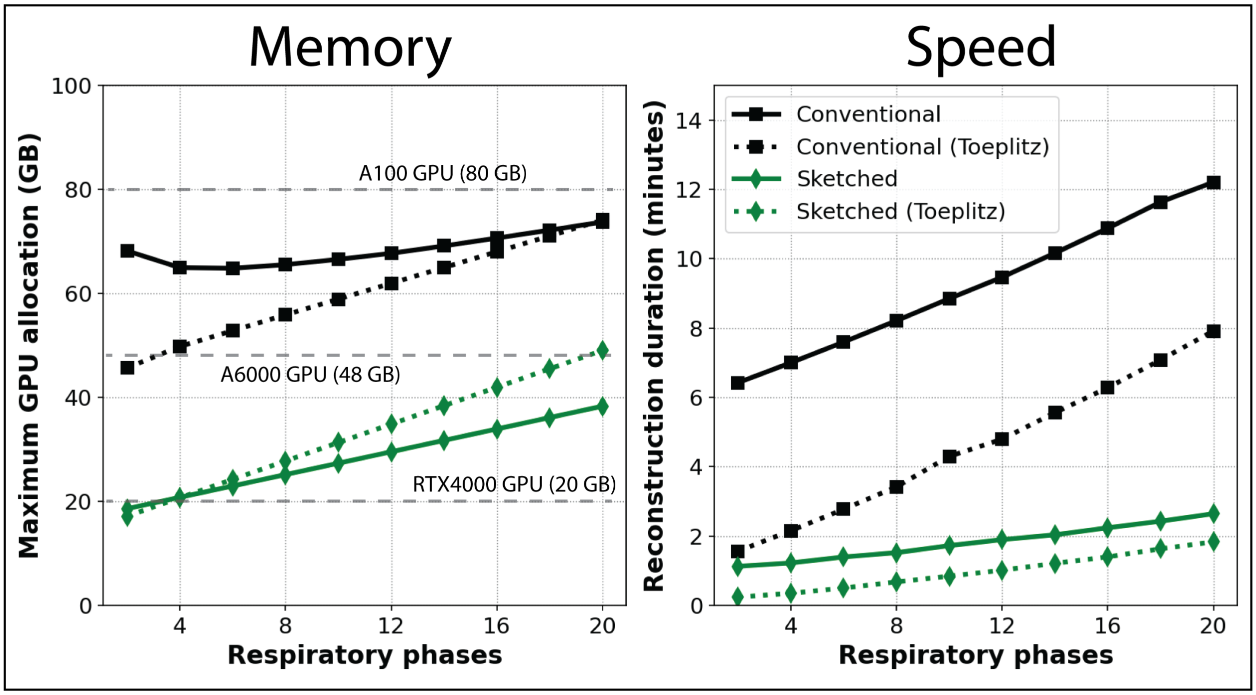

Supporting Figure 2: Memory consumption and iteration speed for conventional, coil sketched (3/15 sketched coils), with and without Toeplitz approximation, for LR reconstructions with 2-20 respiratory phases. Identical settings to the MoCo-LR implementation in Figure 7 are used, but without motion compensation. Commercially available NVIDIA GPUs (A100; A6000; and RTX4000) are shown as examples on the memory plot.
